# Incidence and prognostic factors of primary thyroid lymphoma and construction of prognostic models for post-chemotherapy and postoperative patients: a population-based study

**DOI:** 10.1186/s12902-021-00732-7

**Published:** 2021-04-13

**Authors:** Nan Xiang, Fangyuan Dong, Xuebing Zhan, Shuhan Wang, Junjie Wang, Entao Sun, Bing Chen

**Affiliations:** 1 Department of Pathology, Wannan Medical College, Wenchang West Road 22, Wuhu, Anhui China; 2 Department of Health Inspection and Quarantine, Wannan Medical College, Wenchang West Road 22, Wuhu, Anhui China

**Keywords:** Thyroid lymphoma, Incidence, Treatment, Prognosis, Nomogram

## Abstract

**Background:**

Primary thyroid lymphoma (PTL) is a rare thyroid malignancy, there are few large sample studies on PTL and no standardized treatment regimen has been established due to the rarity. The aims of this study were to explore the incidence and prognostic factors of PTL and construct visual prognostic prediction models for post-chemotherapy and postoperative patients.

**Methods:**

The incidence of PTL in 1975–2017 was extracted from the US Surveillance, Epidemiology, and End Results (SEER) database, then assessed using joinpoint regression software. A total of 1616 eligible PTL patients diagnosed in 1998–2016 were brought into prognostic analysis. Multivariate Cox regression analyses were carried out to reveal independent prognostic elements for overall survival (OS) and cancer-specific survival (CSS).

**Results:**

PTL incidence showed a relatively steady increase in 1975–1994, which annual percent change (APC) was 4.0%, and steady decreasing in 1994–2017(APC − 2.4%). Age, marital status, lymphoma Ann Arbor stage, histological subtypes, surgery, chemotherapy, and radiation were significantly correlated to OS and CSS. Nomograms were constructed to predict OS and CSS in post-chemotherapy and postoperative PTL patients separately, and were verified to have good reliability.

**Conclusions:**

The incidence of PTL increased and subsequently decreased. We revealed the prognostic implications and constructed reliable nomograms for post-chemotherapy and postoperative PTL patients.

## Background

Primary thyroid lymphoma (PTL) is an unusual malignancy, which is defined as an extra-nodal lymphoma confined to the thyroid without any involvement of other areas, comprising approximately 5% of all thyroid malignant tumors and fewer than 3% of all extra-nodal lymphomas, intrinsically related to Hashimoto’s thyroiditis [1]. It is 2–8 times more common in women than in men. Most of the tumors are B-cell-derived non-Hodgkin’s lymphoma; Hodgkin’s lymphoma and T-cell lymphoma are rare [2]. Patients usually show rapid enlargement of the neck mass and may develop hoarseness, expiratory dyspnea, or less commonly, B symptoms such as fever, night sweats, or weight loss [3]. Fine-needle aspiration cytology is not a dependable method for diagnosing PTL, and the main diagnostic method is ultrasound-guided puncture biopsy or surgical biopsy [4]. Combination chemotherapy and radiotherapy has become the standard practice today, surgery alone proved to have recurrence [5]. However, due to the rarity of PTL, no standardized treatment regimen has been established.

Most of the studies on PTL involve case series and case reports, whereas large-scale retrospective population studies are limited. This is currently the largest and most recent prognostic study of PTL patients using the US Surveillance, Epidemiology, and End Results (SEER) database, as the last study of PTL patients using the SEER database was conducted in 2009 by Graff-Baker et al. [6]. In our study, we also explored the incidence of PTL using the SEER database at the beginning, constructed the nomograms for patients after chemotherapy and after surgery, and visualized the prognostic models to achieve the effect that can be used clinically.

## Methods

### Patients

From the SEER database, PTL incidence data from 1975 to 2017 were squeezed out via SEER Stat.8.3.6. Information of 2570 patients with PTL from 1998 to 2016 was extracted to study the prognostic factors of PTL, which is the latest data on PTL cases in SEER Stat 8.3.6. We used histologic codes: International Classification of Diseases for Oncology, 3rd edition (ICD-O-3) histologic codes 9590–9596, 9650–9699, and 9702–9729 to discern lymphoma and site specific code C73.9 to identify lymphoma primarily limited to thyroid. A total of 954 cases with incomplete demographic and clinical information (e.g., race, marital status, surgery record, lymphoma Ann Arbor stage, survival time) were removed, and the remaining 1616 patients were included in the analysis. Then, we screened out patients with PTL after chemotherapy and after surgery, 1074 and 944 eligible patients were included respectively in the post-chemotherapy postoperative patient analysis.

Our analysis included the following demographic and clinicopathological variables: sex, marital status, race, age at diagnosis, year of diagnosis, histologic type ICD-O-3, ICD-O-3 Hist/behav, surgery recode, chemotherapy recode, radiation recode, SEER cause-specific death classification, COD (cause of death) to site recode, survival months, and vital status recode. After screening for incomplete information, the diagnostic year of the remaining 1616 patients ranged from 1998 to 2015, and we divided the cases into three intervals according to the diagnostic year: 1998–2003, 2004–2009, 2010–2015 to see the survival trend of PTL. Race information was classed as black, white, and others (American Indian/Alaskan Native or Asian/Pacific Islander). Age at diagnosis was classified as < 66 years old or ≥ 66 years old, which was based on the median age of 66. When the prognostic factors were analyzed in patients with PTL after chemotherapy, 1074 eligible patients were included in our analysis, age was classified as < 65 or ≥ 65 years old based on the median age of 65. When the prognostic factors were analyzed in postoperative patients, 944 eligible patients were included in our analysis, age was classified as < 64 or ≥ 64 years old, according to the median age of 64. Overall survival (OS) was analyzed from diagnosis to death regardless of any cause, and cancer-specific survival (CSS) was analyzed from diagnosis to death due to PTL. OS and CSS were the primary outcomes.

### Statistical analyses

The annual age-adjusted morbidity of PTL were analyzed per 100,000 persons via SEER Stat.8.3.6. We counted annual percentage change (APC) via the Joinpoint Regression Program 4.8.0.1. Overall survival and cancer-specific survival were calculated using the Kaplan-Meier survival analysis and contrasted via log-rank test. Then we carried out univariate and multivariable Cox regression analyses to reveal independent prognostic factors.

The independent prognostic factors of post-chemotherapy and postoperative PTL patients obtained by Cox regression analysis were included in the construction of nomograms for predicting OS and CSS at 3, 5, and 10 years. We used Harrell’s concordance index (C-index) to assess the nomogram performance, which can calculate the discrepancy between the predicted survival and actual survival. The calibration curves were established to visually evaluate the nomogram performance, which determines whether predicted and actual survival rates were consistent.

All statistical analysis was performed via SPSS 26.0 and R 3.6.3. The R packages involved rmda, survival, rms, ggplot2, survminer, and DynNom. A statistical variation of *P* < 0.05 was considered to be notable.

## Results

### Incidence of PTL

**Fig. 1:**
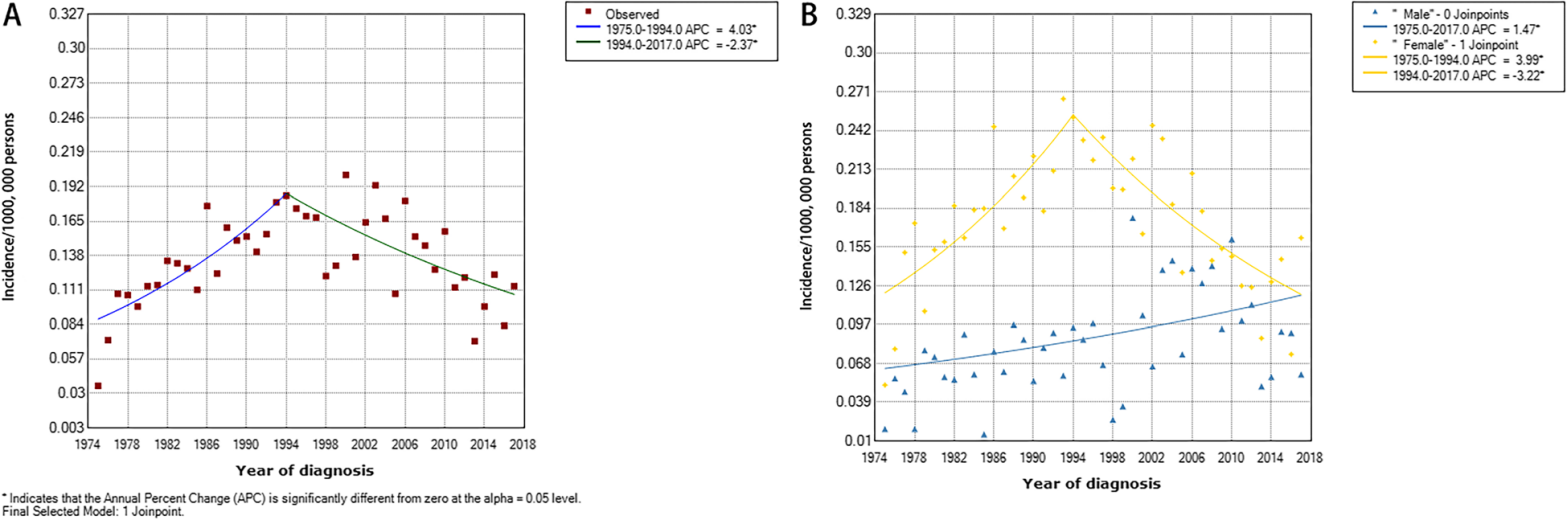
(**a**) Annual age-adjusted incidence of PTL increased in 1975–1994, then decreased in 1994–2017. (**b**) This trend is more pronounced in female population

The survey categorized PTL patients from 1975 to 2017. PTL patients were analyzed to obtain a joinpoint in 1994. Incidence steadily growing from 1975 to 1994, which APC was 4.0% (95% CI 2.0–6.1, *P* < 0.01) and continuously decreased from 1994 to 2017, with an APC of − 2.4% (95% CI -3.5– -1.3, *P* < 0.01) (Fig. 1a). This tendency was more notable in the women (Fig. 1b). The PTL’s yearly age-adjusted occurrence in females was 0.012/100, 000 persons in 1975 and 0.025/100,000 persons in 1994, which APC was 4.0% (95% CI 2.1–5.9, *P* < 0.01). The incidence decreased to 0.012/100,000 persons in 2017, which APC was − 3.2% (95% CI -4.3– -2.1, *P* < 0.01). No joinpoint was obtained for male PTL patients, and the yearly age-adjusted occurrence increased from 0.006/100,000 characters in 1975 to 0.012/100000 characters in 2017, with an APC of 1.5% (95% CI 0.4–2.6, *P* < 0.01).

### Demographics and Clinicopathological characteristics of PTL patients

With an extensive scope of 5–98 years old, the average age at diagnosis was 64.79 ± 15.10. The full cohort consisted of 1111 (68.8%) females and 505 (31.2%) males. The majority of patients were White (89.4%) and married (58.9%). According to lymphoma Ann Arbor Stage, most patients were categorized as Stage I (52.7%). The characteristics of these PTL sufferers are summed up (Table 1).

**Table 1:** Patient characteristics of PTL

Characteristic	No. of patients	Percentage (%)
**Total**	1616	100
**Age at diagnosis**		
Mean ± SD	64.79 ± 15.10	
Median (range)	66.0(5.0–98.0)	
< 66	779	48.2
≥ 66	838	51.8
**Sex**		
Female	1111	68.8
Male	505	31.2
**Marital status**		
Married	952	58.9
Unmarried	664	41.1
**Race**		
White	1445	89.4
Black	36	2.2
Others^a^	135	8.4
**Year of diagnosis**		
1998–2003	469	29.0
2004–2009	614	38.0
2010–2015	533	33.0
**Classification**		
HL	17	1.1
Aggressive B cell NHL^b^	968	59.9
Indolent B cell NHL^c^	477	29.5
T cell NHL	6	0.4
NHL-NOS^d^	102	6.3
Other/Unclassified	46	2.8
**Lymphoma Ann Arbor Stage**		
Stage I	852	52.7
Stage II	564	34.9
Stage III	54	3.3
Stage IV	146	9.0
**Surgery**		
Performed	944	58.4
No	672	41.6
**Chemotherapy**		
Performed	1074	66.5
No	542	33.5
**Radiation**		
Performed	751	46.5
No	865	53.5

^a^ American Indian/Alaskan Native or Asian/Pacific Islander.

^b^ Included DLBCL (diffuse large B cell lymphoma) and BL (Burkitt’s lymphoma).

^c^ Included FL (follicular lymphoma), CLL/SLL (chronic lymphocytic leukemia/small lymphocytic lymphoma), and MALT (mucosal-associated lymphoid tissue)

^d^ NHL-NOS (not otherwise specified)

Because there were too many pathological subtypes of primary thyroid lymphoma, we compiled them separately. All demographic and survival characteristics of PTL sufferers stratified by histological subtype and univariate Cox regression analysis are summarized in Table 2. Based on clinical aggressiveness and cell origin, there were 968 (59.9%) sufferers with aggressive B cell NHL (non-Hodgkin lymphoma), 477 (29.5%) indolent B cell NHL, 102 (6.3%) NHL–NOS (not otherwise specified), and 6 (0.4%) T cell NHLs. The most common histological subtype was diffuse large B cell lymphoma (DLBCL) (57.4%), followed by mucosal-associated lymphoid tissue (MALT) (18.9%), follicular lymphoma (FL) (9.4%), NHL-NOS (6.3%), Burkitt’s lymphoma (BL) (2.5%), Hodgkin’s lymphoma (HL) (2.3%), and finally chronic lymphocytic leukemia/small lymphocytic lymphoma (CLL/SLL) (0.8%).

**Table 2:** Patient characteristics stratified based on histological subtypes

Histology subtypes	n(%)	Median age(range)	Overall survival (OS)				Cancer-specific survival (CSS)			
			Mean OS, m	Median OS, m	HR(95%CI)	P	Mean CSS, m	Median CSS, m	HR(95%CI)	P
All patients	1616	66.0(5–98)	139.04	156.00	/	/	150.58	180.00	/	/
DLBCL	927(57.4)	69.0(13–98)	127.55	133.00	Reference	/	143.25	169.00	Reference	/
MALT	305(18.9)	64.0(16–91)	147.26	195.00	0.57(0.45–0.72)	< 0.001	145.45	175.00	0.77(0.61–0.98)	0.033
FL	152(9.4)	60.5(30–93)	176.98	NR	0.38(0.27–0.54)	< 0.001	170.76	NR	0.55(0.39–0.77)	0.001
NHL-NOS	102(6.3)	70.5(13–95)	118.93	135.00	0.97(0.70–1.33)	0.824	146.68	NR	0.84(0.57–1.23)	0.365
BL	41(2.5)	52.0(18–89)	142.75	NR	0.46(0.24–0.89)	0.022	149.96	NR	0.47(0.22–0.99)	0.047
HL	17(1.1)	27.0(16–74)	191.61	NR	0.19(0.05–0.75)	0.018	191.61	NR	0.24(0.06–0.97)	0.045
CLL/SLL	13(0.8)	76.0(47–92)	92.96	159.00	1.25(0.59–2.63)	0.563	108.62	159.00	1.43(0.64–3.21)	0.384
Others	59(3.7)	67.0(5–98)	110.62	76.00	1.23(0.85–1.78)	0.275	137.89	NR	1.05(0.67–1.65)	0.832

Abbreviations: *HR*, Hazard Ratio; *CI*, Confidence Interval; *NR*, Not Reached; *DLBCL*, Diffuse Large B cell Lymphoma; *MALT*, Mucosal-associated Lymphoid Tissue; *FL*, Follicular Lymphoma; *NHL*, Non-Hodgkin Lymphoma; *NOS*, Not Otherwise Specified; *BL*, Burkitt’s Lymphoma; *HL*, Hodgkin lymphoma; *CLL/SLL*, Chronic Lymphocytic Leukemia/Small Lymphocytic Lymphoma

### Treatments of PTL patients

Beyond half of the patients received chemotherapy (66.5%) and surgery (58.4%). Approximately 46.5% of patients received radiotherapy (Table 1).

In a cohort of patients with PTL after chemotherapy, the median age was 65 years old. In the SEER database, there is no detailed record of chemotherapy methods for PTL patients. In a cohort of patients with postoperative PTL, 344 (36.4%) patients had lobectomy and/or isthmectomy (included lobectomy only, isthmectomy only, and lobectomy with isthmectomy), 136 (14.4%) patients had less than a lobe removed (included removal of less than a lobe, NOS, local surgical excision, and removal of partial lobe only), 120 (12.7%) patients more than a lobe removed (included removal of a lobe and partial removal of the contralateral lobe, subtotal, or near total thyroidectomy), 322 (34.1%) patients underwent total thyroidectomy, and 22 (2.3%) patients had other surgery, NOS. Table 3 summarizes the demographic and survival characteristics of postoperative patients with PTL based on surgical approach.

**Table 3:** Postoperative patient characteristics according to the operation methods

Operation Methods	n(%)	Median Age(range)	Overall Survival (OS)		Cancer-specific Survival (CSS)	
			Mean OS, m	Median OS, m	Mean CSS, m	Median CSS, m
All patients	944	64(13–94)	150.50	176.00	158.69	186.00
Lobectomy and/or isthmectomy^a^	344(36.4)	66(16–94)	147.59	NR	156.80	192.00
Removal of less than a lobe^b^	136(14.4)	65.5(22–92)	125.51	139.00	140.18	167.00
Removal of more than a lobe^c^	120(12.7)	65.5(20–90)	138.53	168.00	152.00	195.00
Total thyroidectomy	322(34.1)	60(13–92)	158.40	199.00	161.37	NR
Others^d^	22(2.3)	66(33–89)	140.44	NR	137.02	186.00

Abbreviations: *NR*, Not Reached

^a^ Included lobectomy only, isthmectomy only, and lobectomy with is thymectomy.

^b^ Included removal of less than a lobe, not otherwise specified (NOS), local surgical excision, and removal of partial lobe only

^c^ Included removal of a lobe and partial removal of the contralateral lobe, subtotal, or near total thyroidectomy

^d^ Surgery, NOS

### Survival analysis of PTL patients

**Fig. 2:**
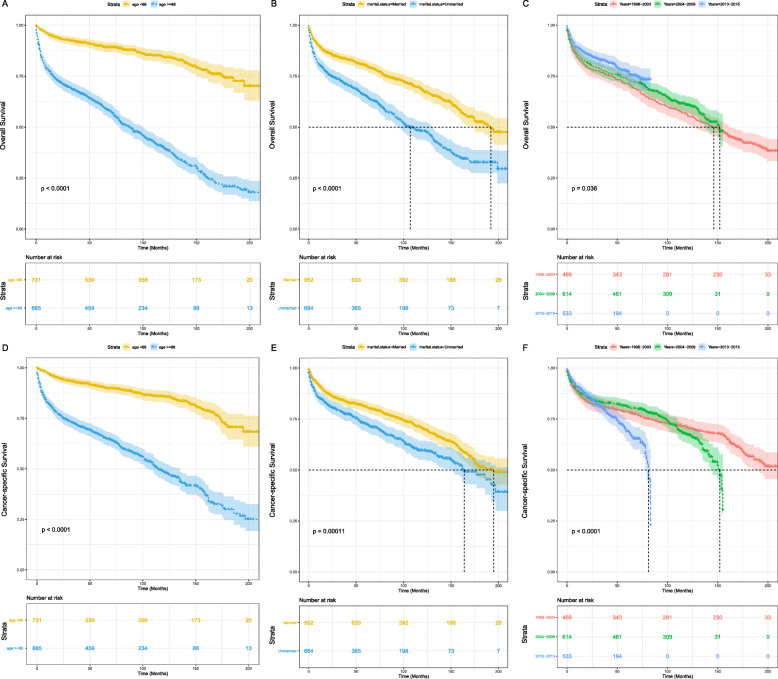
OS analysis of PTL classified by (**a**) age, (**b**) marital status, (**c**) years of diagnosis; CSS analysis of PTL stratified by (**d**) age, (**e**) marital status, (**f**) years of diagnosis

Kaplan-Meier survival assessment was performed based on patients’ age, sex, marital status, race, years of diagnosis, histology subtype, lymphoma Ann Arbor stage, and treatment strategies. The results showed that age was considerably connected with OS and CSS (Fig. 2a and d). Married sufferers tended to possess more considerable OS and CSS than unmarried (Fig. 2b and e). Compared to the patients diagnosed in 1998–2003, the OS improved for sufferers diagnosed in 2004–2009 and for sufferers diagnosed in 2010–2015 (both *P* < 0.05) (Fig. 2c). However, this trend was not observed for CSS, and instead showed the opposite trend (Fig. 2f). In addition, Kaplan-Meier survival analysis presented that there was no notable difference in survival time among genders and races.

**Fig. 3:**
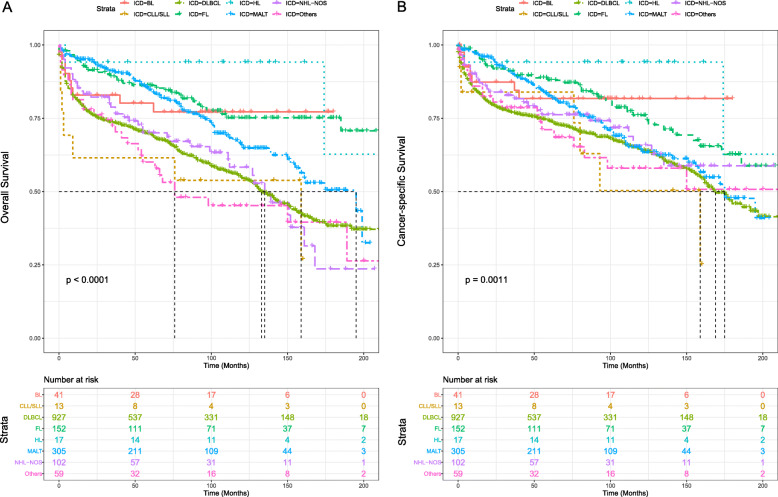
KM survival analysis of (**a**) OS and (**b**) CSS according to the main histological subtypes. (BL, Burkitt’s lymphoma; DLBCL, diffuse large B cell lymphoma; MALT, Mucosal-associated lymphoid tissue; FL, Follicular lymphoma; NHL, non-Hodgkin lymphoma; NOS, not otherwise specified; HL, Hodgkin lymphoma; CLL/SLL, chronic lymphocytic leukemia/small lymphocytic lymphoma)

The mean OS and CSS of PTL patients were 139.04 and 150.58 months, and the median OS and CSS were 156.00 and 180.00 months, respectively. In the cohort of major pathological subtypes, HL had the best mean OS and CSS (both 191.61 months), followed by FL (OS: 176.98 months, CSS: 170.76 months). In addition, MALT had the best median OS (195.00 months). DLBCL, which accounted for the largest proportion, showed a mean OS and CSS of 127.55 months and 143.25 months, respectively. Median OS and CSS were 133.00 months and 169.00 months, respectively. Cox regression model applied to univariate analysis showed that MALT, FL, HL, and BL had better OS and CSS compared to DLBCL (*P* < 0.05) (Table 2). In addition, the OS and CSS’s Kaplan-Meier survival curves for PTL’s chief subtypes are presented (Fig. 3).

**Fig. 4:**
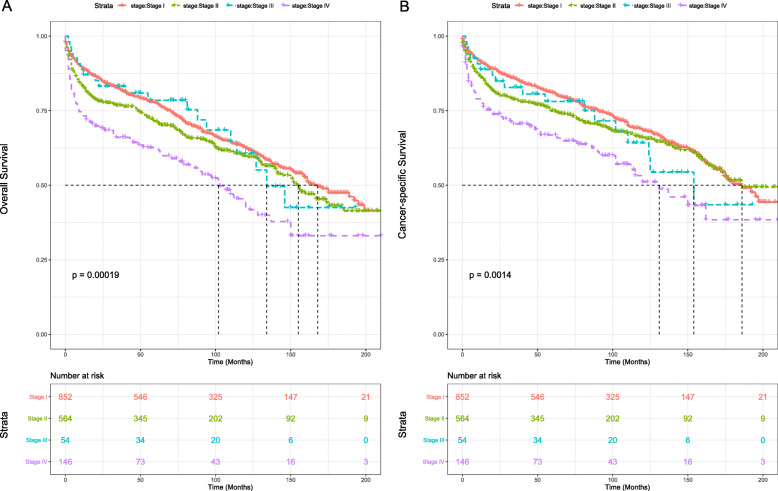
Survival analysis of PTL stratified by Lymphoma Ann Arbor Stage: (**a**) OS, (**b**) CSS

Among different lymphoma Ann Arbor stage cohorts, Kaplan-Meier survival analysis proved that patients with lower stage had significantly better OS (*P* < 0.001) and CSS (*P* < 0.01) than those of higher stage. Stage IV patients showed the worst OS and CSS (Fig. 4a and b).

**Fig. 5:**
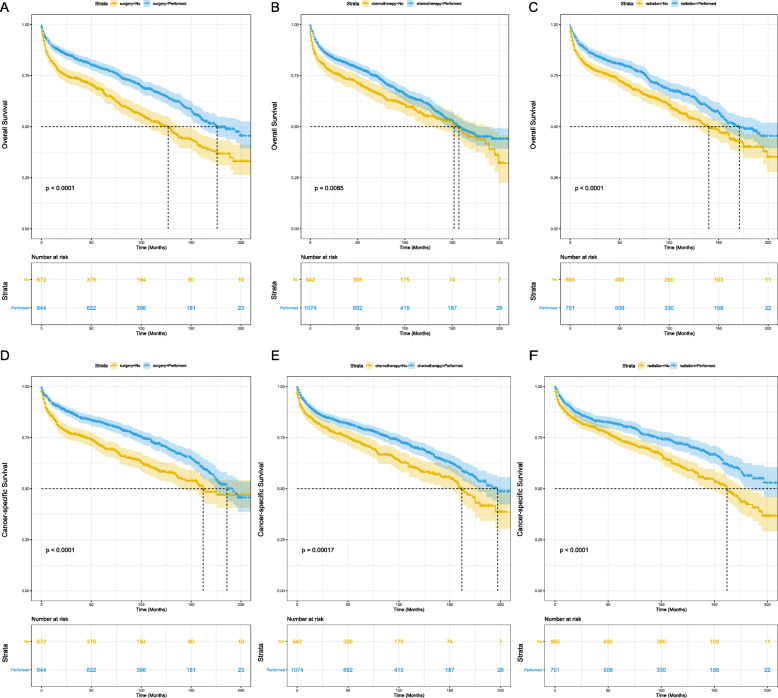
OS analysis of PTL classified by treatment strategy: (**a**) surgery, (**b**) chemotherapy, (**c**) radiation; CSS analysis of PTL classified by treatment strategy: (**d**) surgery, (**e**) chemotherapy, (**f**) radiation

In terms of treatment strategy, we concluded that the OS and CSS of patients with PTL can be significantly improved via surgery (Fig. 5a and d), chemotherapy (Fig. 5b and e), and radiotherapy (Fig. 5c and f).

We carried out multivariate Cox regression analysis to recognize absolute prognostic elements. We revealed that age, years of diagnosis, lymphoma Ann Arbor stage, surgery, chemotherapy, and radiation were significantly correlated with both OS and CSS. In addition, marital status is also an independent prognostic factor for OS (Table 4).

**Table 4:** Independent prognostic factors among PTL patients by multivariable Cox regression analysis

	Overall survival			Cancer-specific survival		
	HR	95% CI	P	HR	95% CI	P
**Age at diagnosis**						
< 66 vs. ≥66	4.611	3.75–5.67	< 0.001	3.976	3.21–4.93	< 0.001
**Marital status**						
Unmarried vs. Married	0.661	0.56–0.78	< 0.001	0.866	0.73–1.04	0.113
**Years of diagnosis**						
1998–2003 vs. 2004–2009	0.941	0.78–1.13	0.520	1.307	1.04–1.64	0.020
1998–2003 vs. 2010–2015	0.705	0.55–0.90	0.005	1.721	1.33–2.23	< 0.001
**Classification**						
HL vs. Aggressive B cell NHL	2.300	0.57–9.29	0.242	1.969	0.49–7.97	0.342
HL vs. Indolent B cell NHL	1.112	0.27–4.56	0.883	1.190	0.29–4.89	0.809
HL vs. T cell NHL	3.438	0.48–24.60	0.219	1.364	0.12–15.16	0.801
HL vs. NHL-NOS	1.965	0.47–8.20	0.354	1.388	0.33–5.89	0.656
HL vs. Other/Unclassified	2.667	0.63–11.39	0.185	2.047	0.47–8.93	0.340
**Lymphoma Ann Arbor Stage**						
Stage I vs. Stage II	1.268	1.06–1.52	0.009	1.292	1.06–1.58	0.011
Stage I vs. Stage III	0.996	0.62–1.59	0.988	1.068	0.66–1.74	0.790
Stage I vs. Stage IV	1.421	1.09–1.85	0.008	1.481	1.11–1.98	0.008
**Surgery**						
No vs. Performed	0.748	0.63–0.89	0.001	0.755	0.63–0.91	0.003
**Chemotherapy**						
No vs. Performed	0.561	0.46–0.68	< 0.001	0.539	0.44–0.67	< 0.001
**Radiation**						
No vs. Performed	0.737	0.62–0.87	< 0.001	0.751	0.63–0.90	0.002

Abbreviations: *HR*, Hazard Ratio; *CI*, Confidence Interval; *HL*, Hodgkin lymphoma; *NHL*, Non-Hodgkin Lymphoma; *NOS*, Not Otherwise Specified

### Prognostic factors analysis of post-chemotherapy PTL patients

At present, chemotherapy is the most commonly used treatment for PTL, so we carried out univariate and multivariate Cox regression analysis to identify the independent prognostic factors in PTL patients after chemotherapy. We included age at diagnosis, sex, race, marriage status, lymphoma classification, lymphoma Ann Arbor stage, surgery, and radiation in univariate Cox regression analysis. The results showed that age at diagnosis, marital status, lymphoma Ann Arbor stage, lymphoma classification, surgery, and radiation were significantly correlated with OS. Age at diagnosis, lymphoma Ann Arbor stage, and radiation were considerably related to CSS.

All considerably dissimilar variables were deduced in the multivariate Cox regression surveys. Four variables (age at diagnosis, marital status, surgery, and radiation) were confirmed as independent prognostic factors of OS. For CSS, we observed that age at diagnosis, lymphoma Ann Arbor stage, and radiation were independent prognostic factors. The consequences of Cox regression analysis of PTL sufferers after chemotherapy are presented in Table 5.

**Table 5:** Independent prognostic factors of OS and CSS in post-chemotherapy PTL patients via univariate and multivariate Cox regression analysis

	(A) Univariate Analysis				(B) Multivariate Analysis			
	Overall Survival		Cancer-Specific Survival		Overall Survival		Cancer-Specific Survival	
	HR (95% CI)	P	HR (95% CI)	P	HR (95% CI)	P	HR (95% CI)	P
**Sex**								
Male vs. Female	1.15(0.92–1.43)	0.213	1.01(0.71–1.43)	0.969	/	/	/	/
**Age at diagnosis**								
< 65 vs. > = 65	4.74(3.72–6.03)	< 0.001	0.26(0.18–0.39)	< 0.001	4.45(3.47–5.69)	< 0.001	3.84(2.60–5.69)	< 0.001
**Race**								
White vs. Black	1.13(0.59–2.20)	0.710	1.46(0.54–3.94)	0.458	/	/	/	/
White vs. Others	1.31(0.91–1.88)	0.147	1.31(0.73–2.38)	0.365	/	/	/	/
**Marital status**								
Unmarried vs. Married	0.60(0.49–0.73)	< 0.001	0.94(0.68–1.31)	0.720	0.74(0.60–0.90)	0.003	/	/
**Classification**								
HL vs. Aggressive B cell NHL	4.47(1.11–17.94)	0.035	1.53(0.38–6.19)	0.551	2.20(0.54–8.91)	0.270	/	/
HL vs. Indolent B cell NHL	2.77(0.67–11.41)	0.158	0.55(0.12–2.50)	0.437	1.52(0.37–6.30)	0.565	/	/
HL vs. T cell NHL	9.54(1.34–67.88)	0.024	3.32(0.30–36.67)	0.328	6.43(0.90–45.87)	0.063	/	/
HL vs. NHL-NOS	5.16(1.23–21.58)	0.025	1.49(0.33–6.80)	0.608	2.15(0.51–9.08)	0.298	/	/
HL vs. Others	5.49(1.22–24.77)	0.027	1.78(0.33–9.74)	0.504	2.81(0.62–12.78)	0.183	/	/
**Lymphoma Ann Arbor Stage**								
Stage I vs. Stage II	0.96(0.76–1.20)	0.730	1.39(0.96–2.01)	0.078	1.07(0.86–1.34)	0.527	1.48(1.02–2.13)	0.038
Stage I vs. Stage III	0.98(0.59–1.63)	0.936	1.41(0.64–3.10)	0.394	0.92(0.55–1.53)	0.736	1.21(0.55–2.68)	0.636
Stage I vs. Stage IV	1.58(1.16–2.15)	0.003	2.20(1.35–3.59)	0.002	1.19(0.87–1.62)	0.279	1.69(1.03–2.77)	0.039
**Surgery**								
No vs. Performed	0.70(0.58–0.86)	< 0.001	0.75(0.54–1.04)	0.085	0.82(0.67–1.00)	0.050	/	/
**Radiation**								
No vs. Performed	0.66(0.55–0.81)	< 0.001	0.56(0.40–0.79)	0.001	0.64(0.53–0.79)	< 0.001	0.56(0.40–0.79)	0.001

Abbreviations: *HR*, Hazard Ratio; *CI*, Confidence Interval; *HL*, Hodgkin lymphoma; *NHL*, Non-Hodgkin Lymphoma; *NOS*, Not Otherwise Specified

### Prognostic factors analysis of postoperative PTL patients

Univariate and multivariate Cox regression analyses were performed by us to reveal the independent prognostic factors and construct nomogram for PTL patients after surgery. We included sex, age at diagnosis, marriage status, race, lymphoma classification, lymphoma Ann Arbor stage, operation methods, chemotherapy, and radiation in the univariate Cox regression analysis. It showed that age at diagnosis, marital status, lymphoma Ann Arbor stage, operation methods, and radiation were significantly correlated with OS and CSS.

All the notable dissimilar variables were deduced in our multivariate Cox regression analysis. Five variables (age at diagnosis, marital status, lymphoma Ann Arbor stage, surgery, and radiation) were confirmed as independent prognostic elements of OS and CSS. The consequences of Cox regression analysis of PTL patients after surgery are shown in Table 6.

**Table 6:** Independent prognostic factors of OS and CSS in postoperative PTL patients via univariate and multivariate Cox regression analysis

	(A) Univariate Analysis				(B) Multivariate Analysis			
	Overall Survival		Cancer-Specific Survival		Overall Survival		Cancer-Specific Survival	
	HR (95%CI)	P	HR (95%CI)	P	HR (95%CI)	P	HR (95%CI)	P
**Sex**								
Male vs. Female	1.13(0.88–1.44)	0.338	1.05(0.80–1.37)	0.741	/	/	/	/
**Age at diagnosis**								
< 64 vs. > = 64	4.91(3.74–6.44)	< 0.001	3.89(2.95–5.14)	< 0.001	4.71(3.57–6.20)	< 0.001	3.83(2.89–5.08)	< 0.001
**Race**								
White vs. Black	1.05(0.50–2.22)	0.900	0.90(0.37–2.18)	0.811	/	/	/	/
White vs. Others	1.02(0.66–1.59)	0.924	0.81(0.47–1.38)	0.435	/	/	/	/
**Marital status**								
Unmarried vs. Married	0.49(0.39–0.61)	< 0.001	0.66(0.52–0.85)	0.001	0.57(0.46–0.71)	< 0.001	0.77(0.60–0.98)	< 0.001
**Classification**								
HL vs. Aggressive B cell NHL	1.91(0.47–7.67)	0.365	1.46(0.36–5.9)	0.594	/	/	/	/
HL vs. Indolent B cell NHL	1.00(0.25–4.08)	0.997	1.05(0.26–4.35)	0.951	/	/	/	/
HL vs. T cell NHL	1.25(0.11–13.83)	0.854	0.00(0.00–575.23)	0.948	/	/	/	/
HL vs. NHL-NOS	2.16(0.50–9.31)	0.302	1.22(0.27–5.59)	0.794	/	/	/	/
HL vs. Others	1.06(0.19–5.78)	0.947	0.79(0.13–4.73)	0.796	/	/	/	/
**Lymphoma Ann Arbor Stage**								
Stage I vs. Stage II	1.28(1.00–1.62)	0.047	1.09(0.83–1.42)	0.532	1.38(1.08–1.75)	0.009	1.16(0.88–1.52)	0.294
Stage I vs. Stage III	0.98(0.50–1.92)	0.955	0.99(0.49–2.01)	0.971	0.68(0.34–1.33)	0.259	0.71(0.35–1.46)	0.353
Stage I vs. Stage IV	1.84(1.25–2.71)	0.002	1.72(1.13–2.63)	0.012	1.67(1.13–2.47)	0.009	1.57(1.03–2.40)	0.037
**Surgery**								
Lobectomy and/or isthmectomy^a^	Reference		Reference		Reference		Reference	
Removal of less than a lobe^b^	1.44(1.06–1.96)	0.020	1.45(1.02–2.04)	0.037	1.59(1.17–2.17)	0.003	1.55(1.10–2.19)	0.013
Removal of more than a lobe^c^	1.25(0.90–1.74)	0.193	1.22(0.85–1.77)	0.286	1.40(1.00–1.95)	0.049	1.37(0.94–1.98)	0.099
Total thyroidectomy	0.79(0.59–1.04)	0.095	0.92(0.68–1.25)	0.602	1.00(0.75–1.33)	1.000	1.08(0.79–1.46)	0.639
Others	0.96(0.45–2.06)	0.917	1.35(0.65–2.77)	0.422	0.90(0.42–1.94)	0.785	1.20(0.58–2.49)	0.631
**Chemotherapy**								
No vs. Performed	0.90(0.72–1.12)	0.342	0.87(0.68–1.11)	0.258	/	/	/	/
**Radiation**								
No vs. Performed	0.78(0.63–0.97)	0.028	0.75(0.59–0.95)	0.019	0.77(0.61–0.96)	0.021	0.74(0.58–0.95)	0.018

Abbreviations: *HR*, Hazard Ratio; *CI*, Confidence Interval

^a^ Included lobectomy only, isthmectomy only, and lobectomy with is thymectomy

^b^ Included removal of less than a lobe, NOS, local surgical excision, and removal of partial lobe only

^c^ Included removal of a lobe and partial removal of the contralateral lobe, subtotal, or near total thyroidectomy

### Nomogram construction and validation

**Fig. 6:**
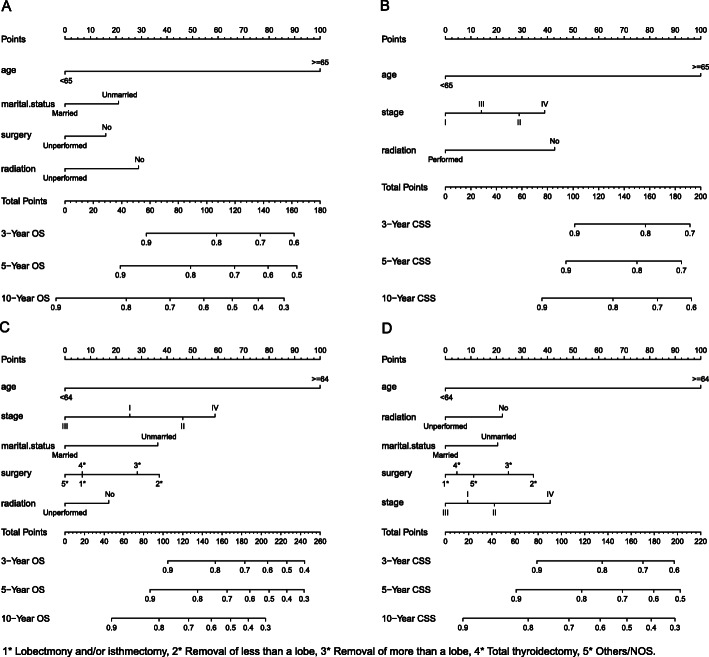
Nomograms for predicting OS (**a**) and CSS (**b**) at 3, 5, and 10 years for post-chemotherapy patients with primary thyroid lymphoma. Nomograms for predicting OS (**c**) and CSS (**d**) at 3, 5, and 10 years for postoperative patients with primary thyroid lymphoma

We constructed two nomograms (Fig. 6a and b) using absolute prognostic elements of OS and CSS respectively among PTL patients after chemotherapy and two nomograms for patients with PTL after surgery, which can predict 3-, 5-, and 10-year OS and CSS (Fig. 6c and d).

We also constructed web page nomograms, which can be viewed online. In the web page, the left option bar can be used for the operator to select the corresponding clinical characteristics of different subgroups independently. Click the “Predict” button, and the corresponding survival rate and its 95% confidence interval results will appear; check the “Predicted Survival at this Follow Up” and drag the “time” button to obtain survival and survival curves at different follow-up times. In addition, we can click on the upper “Numerical Summary” and “Survival Plot” to get the corresponding survival tables and survival curves of patients with different characteristics and follow-up time. For patients after chemotherapy, OS: https://thyroidlymphoma.shinyapps.io/chemotherapy_OS/ and CSS: https://thyroidlymphoma.shinyapps.io/chemotherapy_CSS/.For patients after surgery, OS: https://thyroidlymphoma.shinyapps.io/DynNom_nomogram_PTL_surgery_OS/ and CSS:https://thyroidlymphoma.shinyapps.io/DynNomapp/.

**Fig. 7:**
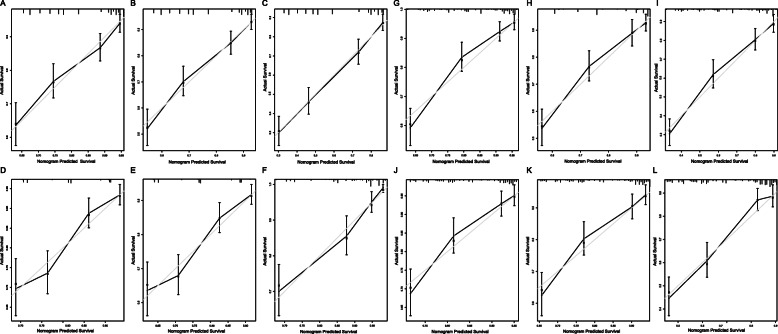
Calibration curves of the nomogram for post-chemotherapy patients for 3-,5-, and 10-year OS (**a–c**) and CSS (**d–f**); Calibration curves of the nomogram for postoperative patients for 3-,5-, and 10-year OS (**g–i**) and CSS (**j–l**)

Harrell’s concordance index (C-index) was calculated to evaluate nomogram performance, and the C-index of the nomograms for OS and CSS values of post-chemotherapy PTL patients were 0.711 (95% CI 0.684–0.738) and 0.707 (95% CI 0.662–0.752), severally. For patients after surgery, the C-index for OS and CSS were 0.743 (95% CI 0.714–0.772) and 0.712 (95% CI 0.679–0.745), respectively. In addition, we calculated the C-index of the nomograms for OS and CSS, which included only lymphoma Ann Arbor stage. The results showed that the C-index were much lower (Table 7). We also established the calibration curves to visually appraise the nomogram performance, which demonstrated good prognostic ability of both OS and CSS nomograms (Fig. 7).

**Table 7:** C-index comparison: prognostic models included in Lymphoma Ann Arbor Stage only vs. our nomograms

		Nomogram		Lymphoma Ann Arbor stage	
		C-index	95%CI	C-index	95%CI
Chemotherapy	OS	0.711	0.684–0.738	0.524	0.493–0.555
	CSS	0.707	0.662–0.752	0.573	0.526–0.620
Surgery	OS	0.743	0.714–0.772	0.552	0.520–0.584
	CSS	0.712	0.679–0.745	0.544	0.509–0.579

Abbreviations: *CI*, Confidence Interval; *OS*, Overall Survival; *CSS*, Cancer-Specific Survival

## Discussion

We observed that the incidence of PTL initially increased from 1975 to 1994 and then decreased to 2017, and this trend was particularly evident in the female population (68.8% of the PTL patients included in our study were female). Studies have shown that similar to Hashimoto’s thyroiditis, thyroid lymphoma incidence in women is 2:1 higher than in men, and even in some studies, it can be as high as 14:1 [5, 7]. The rise and fall in incidence may be related to the continuous improvement and implementation of PTL diagnostic tools, chemotherapy and radiotherapy techniques. Earlier studies have reported that it is difficult to distinguish thyroid lymphoma from undifferentiated thyroid cancer. However, in 1994–2004, the ability to distinguish and diagnose thyroid lymphoma dramatically increased due to new immunocytochemical staining techniques and increased knowledge of cytopathology [8]. This may be the reason why our study showed that 1994 was a turning point in the incidence of PTL. The occurrence of PTL goes hand in hand with chronic Hashimoto’s thyroiditis, studies have shown that chronic autoimmune (Hashimoto’s) thyroiditis has a higher risk of PTL and an increased relative risk of at least 60-fold compared with patients without thyroiditis [9]. It is important to consider the diagnosis of PTL in patients with enlarged neck masses and a history of Hashimoto’s thyroiditis [10]. Clinically, PTL is often used as a differential diagnosis of rapidly enlarging goiter or shortness of breath in patients with thyroiditis, and chemotherapy is started immediately after pathological diagnosis of PTL to avoid airway emergencies [11]. In recent years, some progress has been made in the diagnosis and treatment of Hashimoto’s thyroiditis, which could be conducive to reducing the incidence of PTL [12].

Our study also showed that although female patients had a high incidence of PTL, there was no correlation between gender and prognosis. However, married status is beneficial to the prognosis of PTL patients in our study. Previous studies have shown that marriages with lower lethal cancers (oral and pharyngeal, kidney and renal pelvis, thyroid, and lymphoma) have greater benefits to conditional relative survival, with higher relative survival rates in married patients, and relative survival rates in patients who are separated or divorced or widowed were the lowest [13]. Our study also shows that race has no significant impact on the prognosis of PTL, and no previous studies have reported a correlation between race and PTL prognosis.

The most prevalent type of lymphoma in our study was aggressive B-cell lymphoma, and the most common pathological type was diffuse large B-cell lymphoma (DLBCL), accounting for 57.4% of all PTL pathological types. According to previous case reports, more than 90% of PTL patients present with rapidly expanding goiter, and DLBCL is more aggressive than other PTL subtypes [11]. The second most common pathological type is MALT, an indolent lymphoma, which is a result of chronic inflammation and lymphoplasmacytic infiltration in Hashimoto’s thyroiditis, both of which are closely related [5], a substantial number of patients had thyroiditis history, especially thyroid MALToma. Reports have shown that the prognosis of PTL is associated with pathological subtypes and lymphoma Ann Arbor stage [6, 14, 15]. In our study, different pathological types of lymphoma influence the OS and CSS of PTL patients. The best prognostic pathological type is HL. The prognosis of DLBCL, which accounts for the largest proportion of pathological types, is not as good as MALT in terms of OS or CSS. This is related to the aggressiveness of DLBCL, which grows faster than MALT lymphoma, which often causes local obstructive symptoms, sometimes accompanied by cervical lymph node lesions [15]. In terms of lymphoma Ann Arbor stage, people at lower stages have better prognosis than those at high stages, which is concordant with the findings of a previous study [5].

The current standard chemotherapy regimen is CHOP (Cyclophosphamide, Halotestin, Oncovin, and Prednisone) [16]. DLBCL responds well to chemotherapy, and tumors may shrink within hours after corticosteroid injection [17]. Our results suggest that post-chemotherapy PTL patients treated with radiotherapy have better OS and CSS. In the cohort of chemotherapy patients, age, conjugal status, surgery, and radiotherapy were self-reliant prognostic elements for OS. Surgery and radiotherapy were beneficial for the prognosis of post-chemotherapy PTL patients, while the advantages of radiotherapy were more reflected in CSS. In chemotherapy patients, age, lymphoma Ann Arbor stage, and radiotherapy were independent prognostic factors for CSS. We constructed a nomogram for independent prognostic factors.

In terms of surgical treatment, some studies have shown that chemotherapy and radiotherapy are the mainstream means of PTL treatment, and surgery alone has a certain risk of recurrence [5]. Studies have shown that surgical treatment has a limited effect and that surgery does not significantly improve the OS of patients, which is somewhat inconsistent with our findings that surgery can improve the OS and CSS of PTL [17]. Surgery is rarely beneficial in DLBCL and mixed large cell subtypes because the disease is usually aggressive and disseminated, and it is impossible to surgically remove all lesions; however, thyroidectomy may be associated with great long-term remission and lower incidence [18]. Studies have shown that surgical treatment of PTL patients of the MALT subtype still has good results [8, 14]. The disease-free survival rate of patients with MALT lymphoma treated by surgery alone could be as high as 100% [15, 19]. In consideration of the fact that over half PTL patients in our study had been treated surgically, we also conducted a prognostic study and constructed nomograms of PTL patients who underwent surgical treatment. We reveal that age, conjugal status, lymphoma Ann Arbor stage, surgical procedure, and radiotherapy were independent prognostic factors in the post-operative cohort of patients with PTL.

For PTL patients, our results show that surgery, chemotherapy, and radiotherapy can improve the prognosis of PTL. According to Zhou et al., comprehensive treatment based on radiotherapy or (and) chemotherapy and assisted by surgery can improve efficacy in PTL patients [20]. Multimodal therapy is important for PTL patients, especially those with more aggressive subgroups and extensive local or distant disease; for patients with MALT lymphoma, the benefits of multimodal therapy are less than the risks, which highlights the importance of accurately defining histological subtypes before deciding on treatment options, and the need to consider histology and staging of tumors [10, 18]. Targeted therapies such as rituximab against CD20 on B-cell surface, which is a major advancement in the treatment of lymphoma, have been used in the treatment of DLBCL, but the application of R-CHOP (Retuximab, Cyclophosphamide, Doxorubicin, Vincristine, and Prednisone) therapy in PTL has not been much studied, some literature shows that rituximab has been applied in the treatment of thyroid DLBCL [21–23]. There are also some case reports showing that R-CHOP has a good therapeutic effect on PTL [24, 25]. In recent years, there are also some emerging therapies such as molecular detection, although there is a need to further explore their clinical applications and prognostic effects.

The C-index of the nomograms that were constructed in this study is considerable in both post-chemotherapy and postoperative PTL patients, which is more complete than the prognosis assessed by lymphoma Ann Arbor stage alone, and the calibration curve also reflects the good uniformity between the predicted and actual survival. Of course, our study also has shortcomings. This is a retrospective study, which will lead to some bias in our results, and some potential prognostic factors are missing in the SEER database, or our researchers are not included. Because the dynamic medication status of patients was not included in the SEER database, we could not make use of the fact that advances in medication, such as the molecular targeted drug rituximab, and other effects on prognosis, the patients who were diagnosed with lymphoma too long to draw a single conclusion about the treatment. However, compared with previous studies, our study used SEER database to reveal the trend of PTL incidence rising first and then decreasing, then we investigated the morbidity and prognostic elements of PTL, as well as visualized the prognostic model of post-chemotherapy and postoperative PTL patients for potential clinical application and also provided a certain reference for future research.

## Conclusions

In sum, the incidence of PTL showed an upward and then downward trend, which was most prominent in female patients. Age, marital status, lymphoma Ann Arbor stage, histological types, surgery, radiotherapy, and chemotherapy have certain effects on the prognosis of PTL patients. Younger age, radiotherapy and lower lymphoma Ann Arbor stage, as well as less aggressive lymphoma pathological subtypes, are beneficial to the survival of post-chemotherapy and postoperative PTL patients. We constructed nomograms for their respective independent prognostic factors, and validated that they have good predictive ability.

## Data Availability

Publicly available datasets were analyzed in this study. This data can be found in the SEER database (https://seer.cancer.gov/).

## Abbreviations

APC: Annual Percent Change
BL: Burkitt’s Lymphoma
CHOP: Cyclophosphamide, Halotestin, Oncovin, and Prednisone
CI: Confidence Interval
C-index: Harrell’s Concordance Index
CLL/SLL: Chronic Lymphocytic Leukemia/Small Lymphocytic Lymphoma
COD: Cause of Death
CSS: Cancer-Specific Survival
DLBCL: Diffuse Large B cell Lymphoma
HL: Hodgkin lymphoma
HR: Hazard Ratio
ICD-O-3: International Classification of Diseases for Oncology, 3rd edition
MALT: Mucosal-associated Lymphoid Tissue
NHL: Non-Hodgkin Lymphoma
NOS: Not Otherwise Specified
NR: Not Reached
OS: Overall Survival
PTL: Primary Thyroid Lymphoma
R-CHOP: Retuximab, Cyclophosphamide, Doxorubicin, Vincristine, and Prednisone
SEER: Surveillance Epidemiology and End Results Program
